# Miliary Tuberculosis with Acute Respiratory Distress Syndrome: A Deadly Combination

**DOI:** 10.7759/cureus.33944

**Published:** 2023-01-18

**Authors:** Gaurang M Aurangabadkar, Ulhas S Jadhav, Sumer S Choudhary, Shafee M Khan, Pankaj Wagh, Puja Upadhyay

**Affiliations:** 1 Respiratory Medicine, Datta Meghe Medical College, Datta Meghe Institute of Higher Education and Research, Nagpur, IND; 2 Respiratory Medicine, Jawaharlal Nehru Medical College, Datta Meghe Institute of Higher Education and Research, Wardha, IND

**Keywords:** dexamethasone, antitubercular therapy, hyponatremia, acute respiratory distress syndrome, miliary tuberculosis

## Abstract

A unique and deadly presentation of miliary tuberculosis is acute respiratory distress syndrome. In this case report, we present the case of a 22-year-old male patient who presented with a history of weight loss, appetite loss for eight months, and rapidly worsening dyspnea for 15 days, for which he was admitted to the intensive care unit. Chest X-ray and computed tomography (CT) of the thorax revealed bilateral miliary opacities. Routine blood tests revealed hyponatremia and leukocytosis. The patient was started on non-invasive ventilatory support, intravenous corticosteroids, and anti-tubercular therapy on clinical and-radiological suspicion of miliary tuberculosis. The patient was admitted for one month and started to show rapid recovery after initiating anti-tubercular and corticosteroid therapy.

## Introduction

Gross lymphohematogenous dissemination of the mycobacterium tuberculosis (TB) bacilli leads to a distinct presentation known as miliary TB. Miliary TB is identified through gross pathological examination by the typical presentation of minute tubercles, bearing a striking resemblance to millet seeds both in appearance and size [1].

Miliary TB manifests clinically with non-specific features such as chronic fever, weight, and appetite loss. Productive cough and breathlessness are usual findings in cases of pulmonary involvement. A diagnostic delay usually occurs due to these atypical clinical features. In rare cases, acute respiratory distress syndrome (ARDS) can be a clinical manifestation of miliary TB [2], especially in patients with extensive involvement of the lung parenchyma. The characteristic presentation on chest radiography is a miliary pattern that involves disseminated pulmonary micronodular shadows. In a country with a high prevalence of TB such as India, a high index of suspicion for miliary TB in a patient with non-specific constitutional symptoms is essential to avoid unnecessary delays in diagnosis and treatment initiation.

## Case presentation

A 22-year-old male patient presented to the emergency department with complaints of gradually progressive breathlessness for three months, which had increased significantly over the last 10 days, dry cough, fever, vomiting, and loss of appetite. The patient also reported chronic complaints of left-sided chest pain and weight loss for eight months. Given his worsening oxygenation situation, the patient was admitted to the intensive care unit and started on non-invasive ventilation with oxygen support.

On obtaining the patient’s detailed history, it was revealed that he was well eight months ago before he developed left-sided chest pain, which varied in intensity, and was admitted to a private hospital, where a chest radiograph was conducted and revealed left-sided moderate pleural effusion. Therapeutic thoracentesis was done, and approximately 1000 mL of hemorrhagic pleural fluid was drained. Pleural fluid cytology revealed a lymphocyte-predominant pleural fluid with high cellularity, but no evidence of malignancy was reported by the cytopathologist. The patient had no history of comorbid conditions apart from chronic alcohol consumption for four years.

On general examination, his findings were as follows: pulse rate: 128 beats/minute; respiratory rate: 28 breaths/minute; blood pressure: 90/50 mm Hg; and oxygen saturation (SpO2 %): 88% on room air. Pallor was present, while icterus was absent, and chest auscultation revealed the presence of bilateral crepitations.

**Figure 1:**
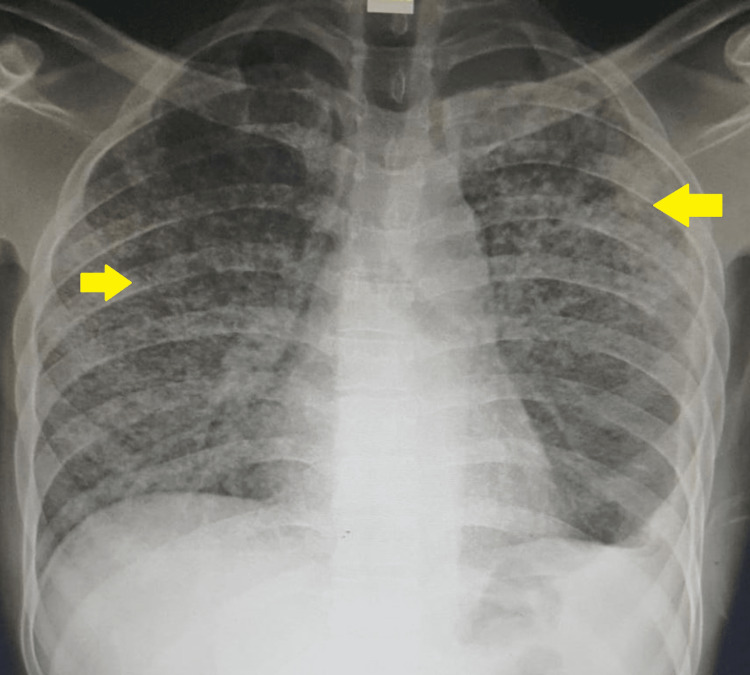
Chest X-ray with a posteroanterior (PA) view on admission showing bilateral symmetrical miliary opacities (yellow arrows).

A chest X-ray with a posteroanterior (PA) view was done on admission and revealed bilateral miliary opacities with symmetrical distribution (Figure 1).

**Figure 2:**
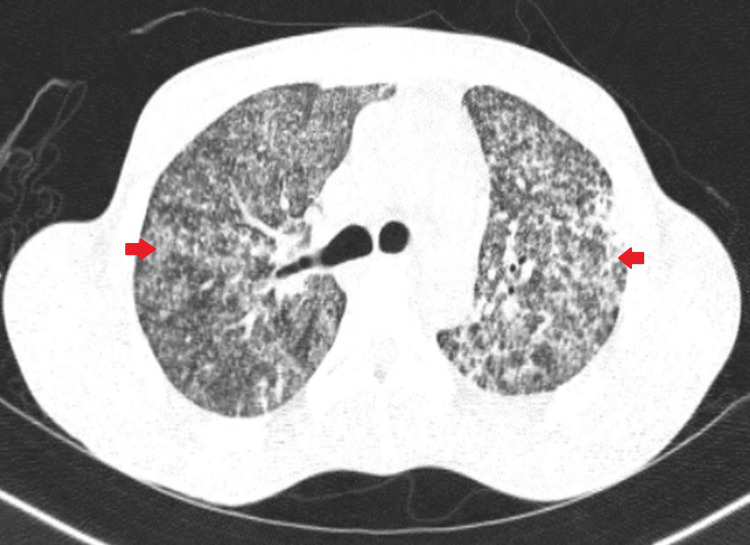
Contrast-enhanced computerized tomography (CT) scan of the chest showing bilateral multiple centrilobular nodules with tree-in-bud appearance (red arrows).

For further evaluation of the abnormal chest X-ray findings, a contrast-enhanced computerized tomography (CT) scan of the thorax was performed, revealing the presence of multiple centrilobular nodules arranged in random distribution with tree-in-bud appearance and lymphadenopathy. The left upper lobe showed consolidation with focal changes of bronchiectasis, and these CT features were suggestive of miliary TB (Figure 2).

For further evaluation, a flexible fiber optic bronchoscopy was done, which showed the presence of thick mucopurulent secretions present in the left bronchial tree. No visible endobronchial mass or anatomical abnormality was observed.

The reports of various other investigations done for the patient are summarized in Table 1.

**Table 1:** Summary of the reports of various other investigations done on the patient.

Investigations done	Reports of the patient
Two-dimensional Echocardiography (2D-ECHO)	Normal study. Left ventricular ejection fraction (LVEF) (%) – 60%, No evidence of pulmonary hypertension.
Sputum for Acid-fast bacilli (AFB)	Negative
Sputum for Xpert® MTB/RIF (GeneXpert) assay	Mycobacterium tuberculosis not detected
Bronchoalveolar lavage (BAL) for AFB	Negative
BAL for Xpert® MTB/RIF (GeneXpert) assay	Mycobacterium tuberculosis not detected
Ultrasonography (USG) of the abdomen	Mild hepatosplenomegaly
Tuberculin skin test (TST)	Positive

After starting non-invasive ventilation (NIV) support and anti-tubercular therapy (ATT), the patient started to show improvement in the oxygenation status and blood parameters and was gradually weaned from oxygen support and shifted to the general ward. 

The patient was discharged after 21 days on ATT with a standard four-drug regimen of Isoniazid (H), Rifampicin (R), Pyrazinamide (Z), and Ethambutol (E), which are to be continued for six months. The patient was asked for a follow-up after seven days but was lost to the follow-up. This loss was probably a result of the considerable geographical distance between the patient’s hometown and our hospital. Hence, no follow-up radiological investigations could be conducted to assess improvements. A differential diagnosis of malignancy was ruled out through a cytological analysis of the pleural fluid and the bronchoalveolar lavage fluid for malignant cells. A proper history ruled out other etiologies such as occupational lung diseases and hypersensitivity pneumonitis.

## Discussion

In the literature, lymphohematogenous dissemination anywhere in the body from the primary focus of TB is commonly referred to as miliary TB; it almost invariably leads to the death of the patient if it goes untreated [3]. In nations with a low-to-moderate incidence of TB, studies demonstrate a mortality of 14% among miliary TB patients who receive treatment [4]. It was also found, however, that the mortality rate increased due to the occurrence of ARDS, multi-organ failure, and septic shock in the patient [4]. Miliary opacities on chest radiography are found in more than 80% of miliary TB patients along with the typical features of TB [5]. Urgent and rapid further diagnostic investigations should be undertaken with the clear aim of initiating early treatment in order to improve the prognosis of the patient.

Non-specific symptoms, such as loss of weight, loss of appetite, chills, and night sweats, and pulmonary symptoms, such as cough with expectoration, breathlessness, and pain in the chest, represent the most common clinical presentations of miliary TB in adults [6]. On general examination of the patient, the clinician may also encounter pulmonary signs such as tachypnea and cyanosis, as well as abdominal findings of hepatosplenomegaly and less common signs such as choroidal tubercles on fundoscopic examination and ascites. A rare presentation of miliary TB can be with ARDS, in less than 8% of patients, and despite the administration of corticosteroids and mechanical ventilation, a high mortality rate, ranging from 40 to 80%, can be witnessed [7].

Diagnostic delay or inability to reach a diagnosis resulting in a subsequent delay in starting anti-tubercular treatment are often implicated as the most significant factors of the high mortality rate of miliary TB patients [7]. Although it is not uncommon to encounter severe complications of miliary TB, early ATT, judicious use of corticosteroids, and access to critical care interventions can drastically lower the mortality in such patients [8,9].

## Conclusions

This case report highlights the condition of a young male patient who had miliary TB, which led to ARDS. Our patient responded well after being aggressively treated for hypoxia and early corticosteroid and anti-TB treatment initiation. Without proper medical care and critical care intervention such as NIV, the patient’s condition would have probably deteriorated rapidly. This highlights the need for aggressive intervention and ATT initiation in a patient with ARDS presenting with radiological findings of miliary TB.
